# The pneumococcal bacteriocin streptococcin B is produced as part of the early competence cascade and promotes intraspecies competition

**DOI:** 10.1128/mbio.02993-24

**Published:** 2024-12-17

**Authors:** J. D. Richardson, Emily Guo, Ryan M. Wyllie, Paul Jensen, Suzanne Dawid

**Affiliations:** 1 Department of Pediatrics, University of Michigan, Ann Arbor, Michigan, USA; 2 Department of Biomedical Engineering, University of Michigan, Ann Arbor, Michigan, USA; 3 Department of Chemical Engineering, University of Michigan, Ann Arbor, Michigan, USA; 4 Department of Microbiology and Immunology, University of Michigan, Ann Arbor, Michigan, USA; The University of Arizona, Tucson, Arizona, USA

**Keywords:** quorum sensing, bacteriocins, *Streptococcus pneumoniae*, genetic competence, interbacterial competition

## Abstract

*Streptococcus pneumoniae* is an important human pathogen that normally resides in the human nasopharynx. Competence-mediated bacteriocin expression by *S. pneumoniae* plays a major role in both the establishment and persistence of colonization on this polymicrobial surface. Over 20 distinct bacteriocin loci have been identified in pneumococcal genomes, but only a small number have been characterized phenotypically. In this work, we demonstrate that three-fourths of *S. pneumoniae* strains contain a highly conserved *scb* locus that encodes an active lactococcin 972-like bacteriocin called streptococcin B. In these backgrounds, the *scbABC* locus is part of the early competence cascade due to a ComE binding site in the promoter region. Streptococcin B producing strains target both members of the population that have failed to activate competence and the 25% of the population that carry a naturally occurring deletion of the ComE binding site and the functional bacteriocin gene. The ComR-type regulator found directly upstream of the *scb* locus in *S. pneumoniae* strains can activate *scb* expression independent of the presence of the ComE binding site but only when stimulated by a peptide that is encoded in the *scb* locus of *Streptococcus pseudopneumoniae*, a closely related bacterium that also inhabits the human nasopharynx. Given the co-regulation with competence and the phenotypic confirmation of activity, streptococcin B represents a previously unrecognized fratricide effector that gives producing strains an additional advantage over the naturally occurring deleted strains during colonization.

**IMPORTANCE:**

*Streptococcus pneumoniae* is a common cause of pneumonia, meningitis, sinusitis, and otitis media. In order to successfully colonize humans, a prerequisite to the development of invasive disease, *S. pneumoniae* must compete with other bacterial inhabitants of the nasal surface for space and nutrients. Bacteriocins are small antimicrobial peptides produced by bacteria that typically target neighboring bacteria by disruption of the cell surface. *S. pnuemoniae* encodes a large number of potential bacteriocin, but, for most, their role in competitive interactions has not been defined. This work demonstrates that isolates that produce the bacteriocin streptococcin B have an advantage over non-producers. These observations contribute to our understanding of the competitive interactions that precede the development of *S. pneumoniae* disease.

## INTRODUCTION

*Streptococcus pneumoniae* resides in the human nasopharynx where it encounters and competes with a diverse collection of *S. pneumoniae* and other members of the nasopharyngeal community. Over time, children tend to be serially colonized with a changing array of pneumococcal strains and often carry more than one strain at any given point in time. This suggests ongoing opportunities for both intra- and inter-species interactions (1–5). Competition for nutrients and space between bacterial inhabitants of the nasopharynx is in part carried out by small antimicrobial peptides called bacteriocins. Bacteriocins often target local bacteria that lack bacteriocin immunity either due to the absence of immunity factors or lack of expression of existing bacteriocin immunity genes (6–8). The pneumococcal genome contains a variety of loci encoding bacteriocins; some are found in all pneumococcal genomes whereas others are only found in a subset (9). The expression of many of the pneumococcal bacteriocins is tied at the regulatory level to the competence state. For example, the universally conserved CibAB bacteriocins play a role in competence-mediated fratricide by targeting members of the pneumococcal population that have failed to trigger competence and therefore are not producing the immunity protein CibC (10). CibAB expression has been shown to play a role in protecting a resident population of *S. pneumoniae* against invasion during the early stages of colonization in the mouse model (11). The production of *blp*-encoded bacteriocins is indirectly tied to competence via secretion of the *blp*-inducing peptide, BlpC, by the ComAB transporter (8, 12, 13). The *blp* locus is found in all genomes, but unlike the *cibABC* locus, the bacteriocin/immunity content is variable from strain to strain allowing for intra-species competition that is independent of the activation state of the neighboring strain. We have shown that the *blp* locus plays a role in intraspecies competition *in vivo*, especially in the subset of isolates that encode a dedicated BlpAB transporter (7, 8, 12, 14, 15). Isolates with an intact BlpAB transporter can both activate the *blp* locus independently of competence and are characterized by more permissive activation of competence due to the secretion of competence-stimulated peptide (CSP) by BlpAB. The variable content and expression of bacteriocins in any individual isolate play a significant role in dictating the outcome of any interaction with the resident community. Of the remaining 20 or more described bacteriocin loci found in pneumococcal genomes, only the *blp* bacteriocins, CibAB, and the locus encoding Pneumolancidin K have phenotypic confirmation of their inhibitory activity (7, 10, 15–17).

In this work, we characterize the regulation, function, and distribution of one of the five known *S. pneumoniae*-encoded lactococcin 972-like bacteriocins called streptococcin B. Of the five described loci, streptococcins A, B, and C are found in nearly all strains, whereas streptococcins E and D are less common (9). Prior to this work, no activity had been attributed to any of the lactococcin 972-like loci in *S. pneumoniae*. Here, we confirm that a version of the *scb* locus encoding streptococcin B is found in nearly all pneumococcal genomes and, in a majority of isolates, is a previously unrecognized component of the early competence regulon. A subset of strains including the common laboratory strains Tigr4, D39, and R6 lack the competence regulatory region as well as the functional bacteriocin peptide. These strains do not appear to endogenously activate the locus including the dedicated immunity proteins under any tested growth conditions. However, *scb* expression does occur in these strains via the locus-associated ComR homolog, but only when stimulated by a comX-inducing peptide (XIP) encoded within the homologous *scb* locus of *Streptococcus pseudopneumoniae*. D39-like strains are inhibited by isolates expressing the fully functional locus in plate assays and during biofilm growth under competently permissive conditions. Streptococcin B is an additional member of the previously characterized fratricide effectors, allowing for inhibition of both non-competent and streptococcin B negative neighbors.

## RESULTS

### Characterization of the variability of the *scb* locus in pneumococcal genomes

**Fig 1:**
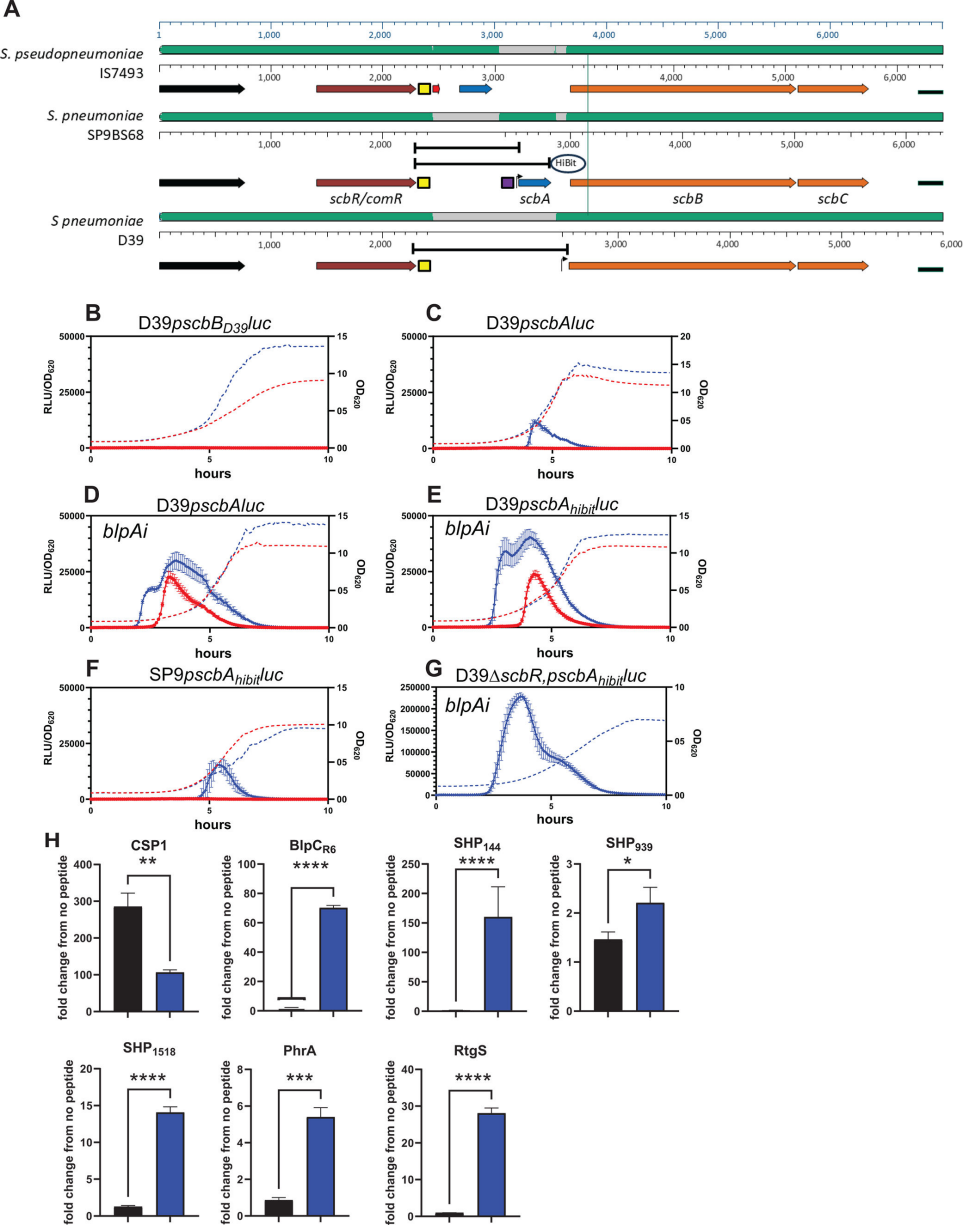
Diagrammatic representation of the *scb* loci and activation characteristics. (**A**). Mauve alignment of the D39, SP9BS68, and *S. pseudopneumoniae scb* loci. ORFs are depicted as arrows; binding sites, as boxes. The ComR homolog encoding gene referred to as SPD_1786 in D39 is shown in brown, the *scbA* gene encoding the functional bacteriocin is shown in blue, and the genes encoding the ScbB and C immunity and transport proteins are shown in orange. The putative XIP-like ORF *scbS* is identified in *S. pseudopneumoniae* and is shown as a red arrow. The ComE binding site and ComR binding sites are shown as purple or yellow boxes, respectively. Areas of homology to the *S. pseudopneumoniae* sequence are shown as green shading, and areas lacking homology are gray. Transcriptional start sites determined either by the pneumoexpress database (D39) or expected start sites based on distance from the ComE binding site (SP9) are shown as bent arrows. The promoter regions cloned into promoters with or without the Hibit tag are shown as bracketed black lines. (**B–G**). Promoter activity of the various *sbc* promoter-luciferase fusions during growth in CDM at pH 7.4 (blue) and pH 6.8 (red). RLU/OD_620_ is shown on the left Y axis and represented by solid lines, whereas OD_620_ alone is shown on the right Y axis and represented by dotted lines. *blpAi* denotes strain backgrounds with an intact *blpA* ORF. (**H**). Promoter activation of either the D39*sbcA* reporter strain (black bars) or the peptide-specific reporter strain (blue bars) 45 min following the addition of 250 ng/mL of the peptide. Activation is shown as fold change from the same reporter with vehicle-only addition. All reporters in H were created in the D39 background (*blpA* deficient).

In the context of understanding the contribution of bacteriocin loci controlled by peptide pheromone-based quorum sensing systems, we focused on the *scb* locus previously described by Rezaei Javan et al. (9). The *scb* locus was predicted to encode a lactococcin 972-like bacteriocin called streptococcin B in addition to ScbB and ScbC, which are predicted immunity and transporter proteins, respectively, although the function of the B and C gene products has not been experimentally verified in any lactococcin 972 loci. The *scb* locus was found directly downstream of a previously uncharacterized ComR-type transcriptional regulator, suggesting a role in the regulation of the locus. We refer to this regulator as ScbR. Rezaei Javan et al. found the *scb* locus in all 571 genomes queried but with variable completeness. Of the 571 genomes, 414 (72.5%) were found to have a complete *scbABC* operon, whereas 157 (27.5%) only encoded the non-bacteriocin proteins ScbBC (Fig. 1A). To better understand the contribution of this locus to pneumococcal competition, we created a series of reporter strains where the *scbBC* promoter region from D39 or the *scbABC* promoter region from the SP9BS68 strain were joined to a luciferase gene and cloned this into the transcriptionally silent CEP locus in a D39 background created either with or without an intact *blpA* gene. The regions cloned into reporter backgrounds are shown in Fig. 1A, a more detailed map is provided in Fig. S1. We additionally created a reporter that incorporated the HiBiT tag into the C terminal end of the streptococcin B peptide with the luciferase gene placed in operon structure after the *scbA* gene (*scbA_HiBiT_*) to assess secretion of streptococcin B. These reporter strains were initially grown in chemically defined media at pH 6.8 and 7.4 to examine pH-mediated differences in expression. The D39-derived *scbBC* promoter did not appear to activate under any of these conditions (Fig. 1B), but the SP9BS68 *scbABC* promoter activated at pH 7.4 in a *blpA*-deficient background (Fig. 1C) and at both pH 6.8 and 7.4 in the background of a *blpA*-sufficient strain (Fig. 1D). There was no difference in activation when comparing the promoter-only reporters with the *scbA_HiBiT_* containing constructs (Fig. 1D and E). Additionally, the reporter created in the native SP9 background behaved similarly to the D39 reporters in a *blpA* deficient background, consistent with the known disruption of *blpA* in this strain (Fig. 1F).

### Activation of the *scbABC* locus by CSP and *S. pseudopneumoniae* XIP

We initially attempted to activate the *scbABC* locus using 250 ng/mL of a 10 AA version of a hypothesized XIP-type peptide encoded in both the *S. pneumoniae scbBC* and *scbABC* loci (SYQNSSKVRL) (Fig. S1). However, addition of this peptide did not activate either reporter construct (not shown). To determine if other, previously described, peptide signals were involved in *scb* control, 250 ng/mL of the peptides SHP143, SHP939, SHP1518, PhrA, RtgS, BlpC2, and CSP1 were used to induce expression of the *scbABC* reporter strain. Control reporters were used for each peptide in the D39 background to verify induction of peptide-responsive promoters (Fig. 1H). In each case, peptides induced their respective control reporters but only CSP1 induced the expression of both the control reporter and the SP9BS68-derived *scbABC* promoter. Addition of CSP1 did not induce expression of the D39-derived *scbBC* promoter (not shown).

**Fig 2:**
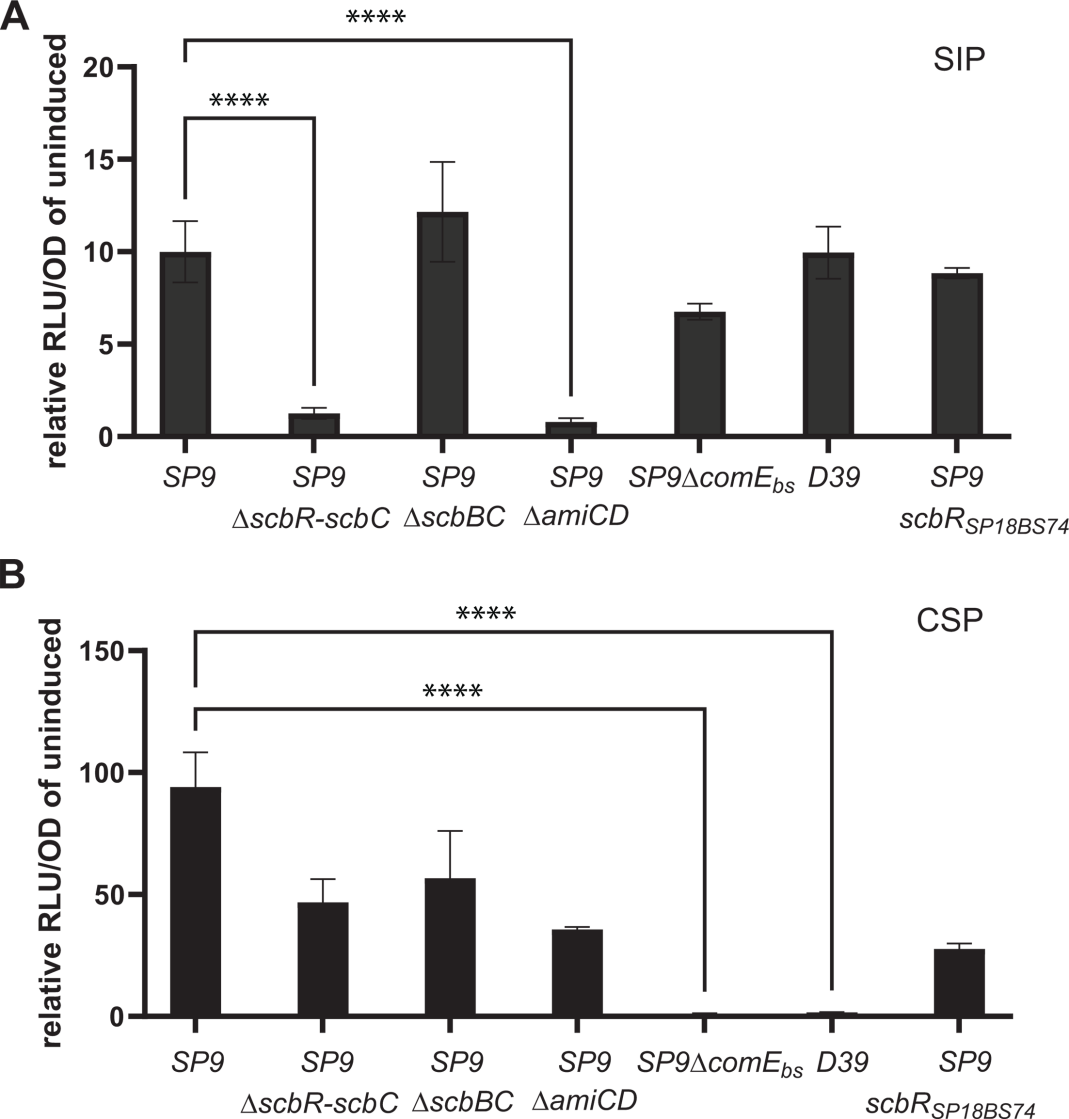
Role of the *scb* locus in regulation of streptococcin B production. D39 strains with the marked p*scb* reporters were grown in CDM 6.8 and induced with 250 ng/mL of S. pseudopneumoniae SIP peptide (**A**), CSP (**B**), or vehicle control at 37°C. Luminescence/OD values were determined after 30 min of incubation and compared with the no peptide control for each reporter. Each reporter strain is designated on the graph by the origin of the promoter content on top (SP9, SP9 with ComE binding site deletion, or D39) and any additional chromosomal mutation below. Reporter strains were tested in the D39 *blpA* intact background and compared by one-way ANOVA for significance.

In surveying other streptococcal species for the presence of *scbA* homologs, we identified a *S. pseudopneumoniae* strain (SPPN IS7493) with a highly homologous *scbABC* locus (Fig. 1A; Fig. S1). The *S. pseudopneumoniae scbABC* locus also contained an upstream ComR homolog, ScbR. By comparing the nucleotide sequence downstream of the *scbR* and upstream of the *scbBC* or *scbABC* locus, we identified a canonical ComRS promoter motif—taag**gacat**tg(**a/g)tgtc**cttg-n_20_-**tataat**—in *S. pseudopneumoniae* as well as in SP9-type and D39-type strains of *S. pneumoniae*. Interestingly, only in *S. pseudopneumoniae* were we able to identify the expected cognate *comS* gene downstream of the promoter motif (here named *scbS*). The *scbS* gene encodes a short 19-AA peptide with the canonical type II XIP double tryptophan motif: MFGFIMFLTYFSF**GDWWHG** (here referred to as treptococin inducing peptide [SIP]). Based on the well-characterized ComRS systems in *Streptococcus mutans*, we predicted that the mature SIP sequence would be the final six C-terminal amino acids. SIP was synthesized and tested against the reporter strains. The six AA SIP peptide successfully activated both the D39 *scbBC* and SP9 *scbABC* promoters (Fig. 2A). Consistent with previously characterized ComRS systems, this stimulation is dependent on an intact AmiCDE importer. No stimulation was noted in a *scbR* deletion background (Fig. 2A), suggesting that this regulator interacts with the peptide. A survey of the pneumococcal SP9 and D39 genomes does not reveal any region that would be predicted to encode this peptide. These data suggest that the *scb* locus can be regulated by the ComR like regulator found adjacent to the rest of the *scb* gene cluster when stimulated with an exogenously supplied XIP-type peptide.

### Verification of a ComE binding site in the *scbABC* promoter

Given the robust stimulation of the *scbABC* locus by CSP1, we examined the promoter for a ComE or ComX binding site. A classic ComE binding site (catttcagggtgctattttgacaatttag) was identified in the *scbABC* promoter 115 bp upstream of the start codon of *scbA* (Fig. 1A; Fig. S1). This sequence was not found in the D39-derived *scbBC* promoter region. Alignment of these two *scb* loci demonstrated that the deletion that resulted in loss of the *scbA* gene in D39 also included the region of the promoter that contains the ComE binding site (Fig. 1A; Fig. S1). To demonstrate the role of the ComE binding site in CSP-mediated regulation of the *scbABC* locus, we created a reporter strain where just the ComE binding site was deleted. As expected, this strain no longer activated luciferase expression in response to addition to CSP (Fig. 2B), although activation with the *S. pseudopneumoniae* SIP was retained (Fig. 2A). To determine if the upstream ComR homolog, ScbR, played a role in competence-mediated control of the *scb* locus, we created a deletion of the entire *scb* region including the ComR regulator gene in our existing reporter backgrounds. These reporters had identical *scbABC* regulation compared with intact backgrounds when allowed to induce naturally (Fig. 1G) or when stimulated with CSP (Fig. 2B), indicating that CSP-induced activation of *scb* is independent of ScbR. Retained natural activation of *scbABC* in the *scbR* deletion strains (Fig. 1G) additionally suggests that, unlike what is found in other Strep species, *this comR* homolog is not required for the activation of competence. Alignment of the *scbR-scbABC* intergenic region from the *S. pseudopneumoniae* strain (Fig. 1A) demonstrates that the region encoding the SIP peptide was likely exchanged for the ComE binding site at some point early in pneumococcal evolution. These data demonstrate that in *scbA* containing strains, the *scb* locus is activated by both a ComR/XIP system and the traditional competence system via ComE binding and activation. D39-like loci are missing the ComE binding site, hence activation of the locus is not tied to the competence system in these backgrounds; however, the ability to respond to SIP is retained. The stimulation of the *scbA* reporter strains is nearly 10× higher following the addition of CSP compared with SIP. Given this, we cannot eliminate the possibility that the peptide is not optimized to work with the *S. pneumoniae* system; however, SIP does stimulate the locus above that seen in wells without peptide addition. These data demonstrate that an *scb* locus that encodes the active ScbA peptide can be stimulated by both an XIP-type peptide and CSP, whereas the locus that is missing *scbA* is only activated by the XIP-type peptide.

The D39 *scbBC* region was included in the transcriptome analysis done by Aprianto et al. (18). In their accompanying searchable database, Pneumoexpress (https://veeninglab.com/pneumoexpress-app/), both *scbB* (SPV_1785) and *scbC* (SPV_1784), show 2-fold to 3-fold increases in average transcript following either 30 or 60 min of exposure to live A549 cells. In an attempt to determine if the D39 locus might induce in response to factors secreted by epithelial cells, we reproduced the cell exposure as described by Aprianto using the D39 strain with the P*_scbBC_* reporter and compared activation of the reporter with cells in the medium alone. This strain did not show any evidence of activation following exposure to epithelial cells (Fig. S2).

### Contribution of *blpA* status to the activation of the *scbABC* promoter

**Fig 3:**
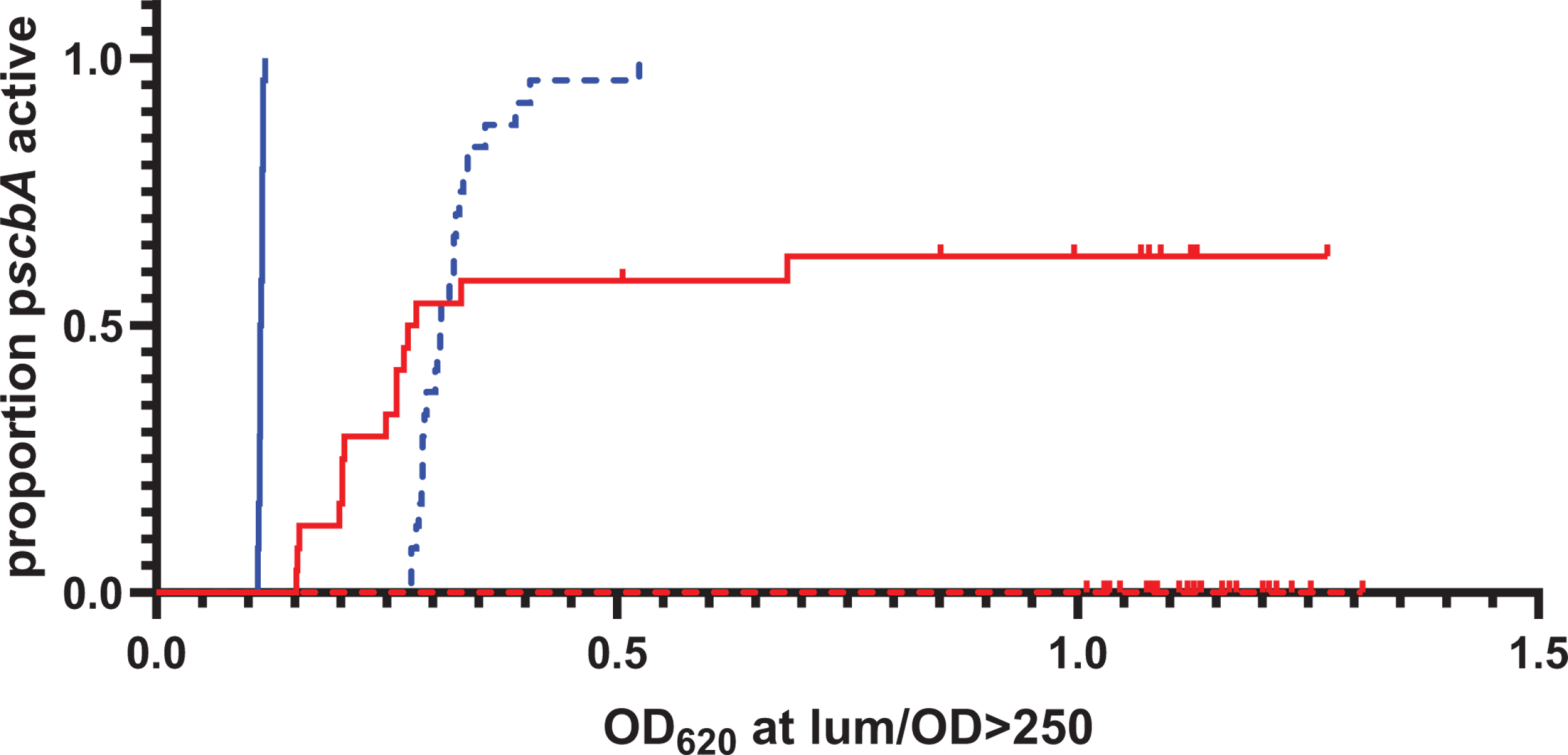
BlpA intact strains activate *scbABC* promoter at low and high pH. The *scbABC* reporter was grown in CDM at pH 6.8 (red) and 7.4 (blue) in the *blpA* intact (solid) and frame-shifted (dashed) backgrounds. Twenty-four wells were used to assess each strain/condition in a 96-well plate. The OD was noted when an individual well exceeded the threshold of 250 RLU/OD_620_.

The status of the bacteriocin encoding *blp* locus can have a substantial impact on competence development. Strains that encode an intact BlpAB transporter induce competence earlier in the growth phase and under typically non-permissive conditions. This is due to the secretion of CSP through the BlpAB transporter, augmenting its extracellular accumulation (8, 19). To determine how BlpA intact strains induce the intact *scbABC* locus differently than those that lack a functional BlpAB, we allowed identical *scb* reporter strains created in otherwise isogenic *blpAB* intact and Δ*blpA* backgrounds to grow in CDM at competence non-permissive pH 6.8 and competence permissive pH 7.4 and monitored individual wells for activation. Under these conditions, only *blpAB* intact strains activated uniformly at pH 6.8 and 7.4 (Fig. 3). As expected, the Δ*blpA* strain did not activate in competent non-permissive pH conditions. This background activated only ~60% of wells at pH 7.4; however, activation in this case occurred at higher ODs compared with *blpAB*-intact strain at the same pH. These data suggest that *blpA*-sufficient strains with the SP9-like *scb* locus will activate under a wider array of conditions compared with *blpA*-deficient backgrounds, potentially providing a competitive advantage in this background.

### Distribution of the *com* activated *scbABC* locus in a defined collection of pneumococcal genomes

**Fig 4:**
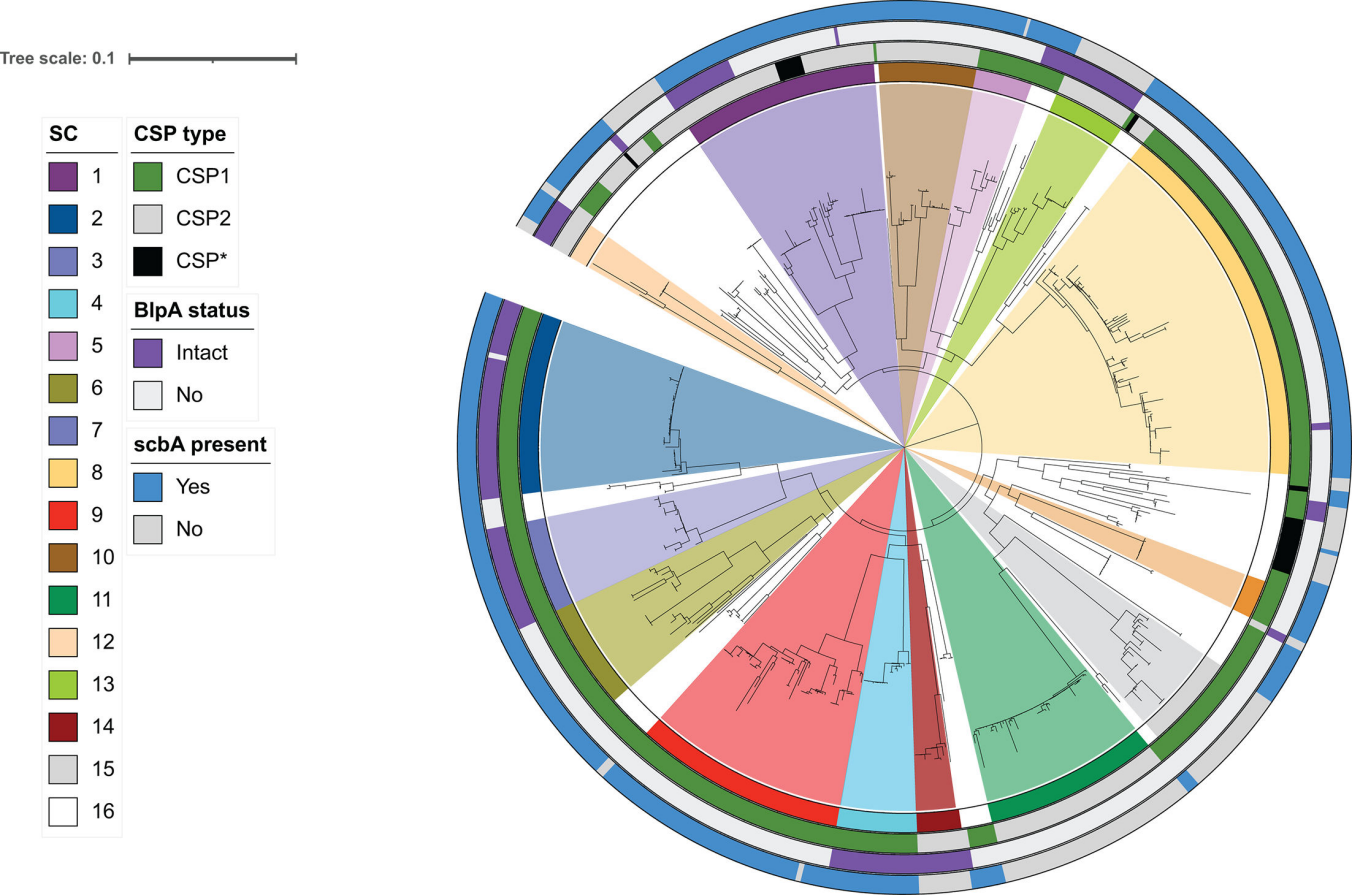
Distribution of the SP9 and D39 like versions of the *scbA* locus in the Boston isolate collection. Genomes were searched for SP9 *scbA* sequences in addition to *blpA* and *comC* sequences using the PubMLST website. SC designations assigned by Mitchell et al. (19) are shown in colored wedges. *blpA*, *scb,* and CSP content is noted in outside rings. 15 SC were defined by this group; SC 16, noted by white wedges, is a collection of ungrouped isolates. The tree was recreated using the Newick file provided by Mitchell et al. using iTOL.

We surveyed the 617 genomes analyzed by Mitchell et al. consisting of 15 different strain clusters (SC) to determine the distribution of competence regulated *scb* loci (Fig. 4). Consistent with the findings of Rezaei Javan et al., we found that 150 genomes (24%) had the D39-like *scbBC* locus and 466 (76%) had the SP9-like *scbABC* locus. All genomes were predicted to encode an ScbR regulator upstream of *scb*. Eighty-four of 617 genomes encode an allelic variant of the more common SPR_1786 *scbR*. This variant has 73% similarity and 57% identity to SPR_1786 with the majority of the identity in the N terminal 103 AA (101/103 identity). This variant was cloned from the pneumococcal strain SP18BS74 and moved into the D39 reporter background replacing the native *scbR* gene. This reporter was shown to also respond to *S. pseudopneumoniae* peptide (Fig. 2A). All 466 SP9-like loci had the identified ComE binding site. The full length *scbABC* locus was found in 11 of 15 clustered SC, whereas the *scbBC* locus was found in 7 of 15 clustered SC. There was no absolute correlation between CSP type and the competence regulated version of the locus; CSP1 encoding strains made up 358/466 (77%) of the *sbcABC* strains compared with 396/617 (64%) total strains. Considering the difference in activation of the *sbcABC* locus noted in *blpA* intact vs disrupted backgrounds, the distribution of BlpA expressing strains was examined. Of the 179 genomes with a predicted intact *blpA* gene, 139 (77%) are predicted to encode *scbA*, similar to the distribution in the whole population. These data demonstrate the broad distribution of the SP9-like version of the *scb* locus in the pneumococcal population. In addition, despite enhanced activation in *blpA* sufficient backgrounds, SP9-like loci are not found more often in these backgrounds. It is possible that there is a trade-off between enhanced competence activation and fitness that limits the *blpA* sufficient population.

### Streptococcin B is not secreted by BlpAB or ComAB

**Fig 5:**
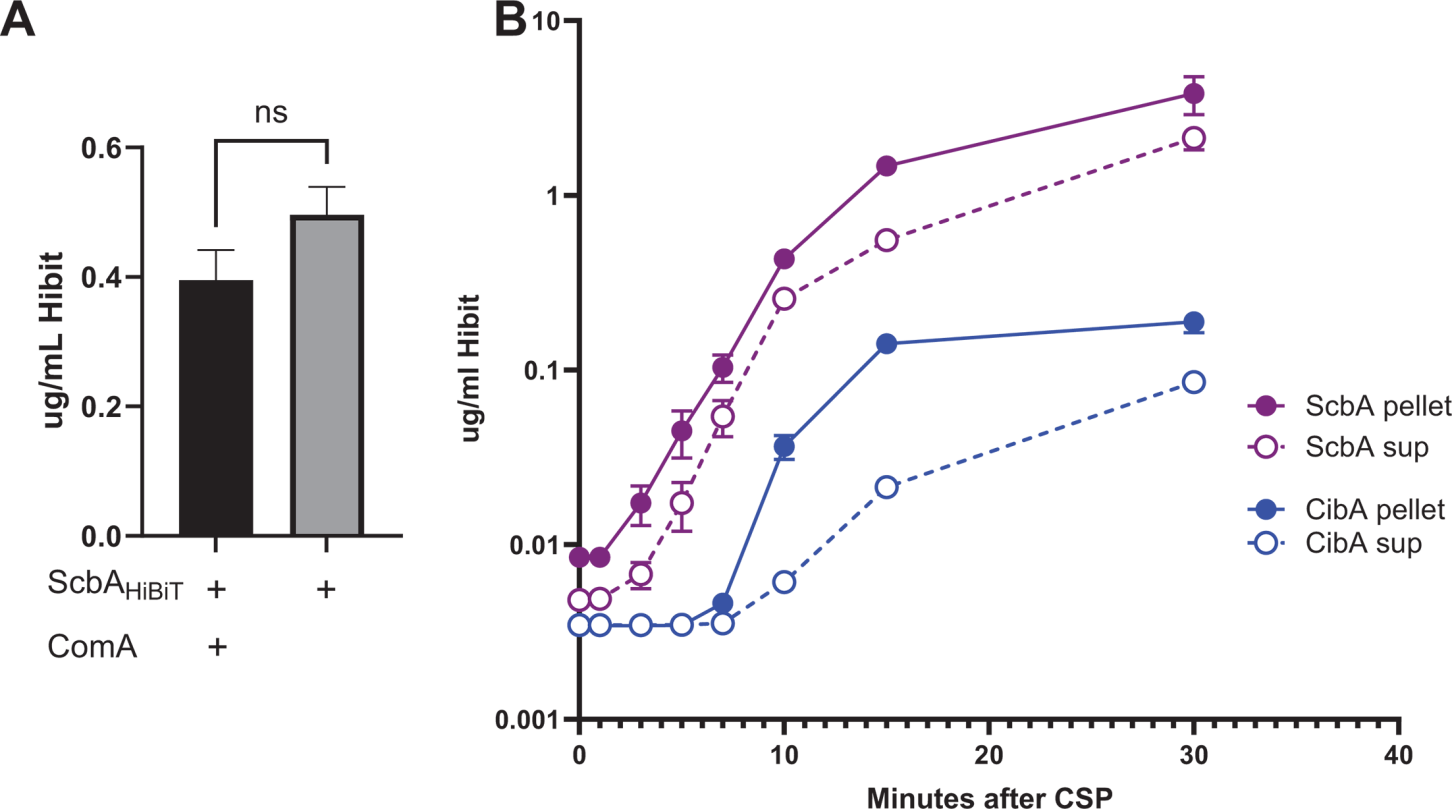
Secretion of ScbAHiBiT is independent of BlpAB and ComAB and occurs early in competence. (A) D39 reporters with ScbAHibiT fusions were grown in CDM and induced with CSP in a *comA*-sufficient and deleted background. Following 1 h of stimulation, the supernatants were assessed for the concentration of the HiBiT tag. Quantification was determined using dilutions of the idealized HiBiT peptide. (B) ScbHiBiT or CibAHiBiT reporter strains were stimulated with CSP at time = 0. Samples were removed at 0, 2, 5, 7, 10, 15, and 30 min and centrifuged; the supernatants and pellets were placed on ice. The resultant samples were assayed for HiBiT concentration using a standard curve generated with the HiBiT standard peptide.

Lactococcin 972-like bacteriocins are thought to be secreted by the Sec system and do not require a specialized processing and secretion system like the Cib and Blp bacteriocins. Given the regulatory connection to competence stimulation, we investigated whether the ComAB transporter was required for streptococcin B secretion. Using the reporter expressing a HiBiT-tagged version of streptococcin B, we assayed supernatants for HiBiT tag secretion following CSP induction in strains expressing or lacking the ComAB transporter. Of note, the D39 background naturally lacks other potential bacteriocin transporters including RtgAB and BlpAB. HiBiT-tagged streptococcin B was secreted into the culture supernatant following CSP stimulation in both ComAB containing and lacking backgrounds in support of secretion via the Sec system (Fig. 5A).

### Streptococcin B is produced earlier and is highly secreted compared with CibA

To compare the secretion kinetics of streptococcin B with that of the fratricide effector bacteriocin, CibA, which is encoded by a late competence gene and dependent on ComAB secretion, we performed a time course experiment using otherwise isogenic strains expressing either CibA-HiBiT or streptococcin B-HiBiT. Streptococcin B-HiBiT was detectible in the supernatant in as little as 3 min post-stimulation, whereas CibA secretion was delayed in comparison, detected between 7 and 10 min following CSP stimulation (Fig. 5B). A larger percentage of HiBiT-tagged Cib remained cell associated compared with streptococcin B. Although we cannot exclude differential effects on secretion brought about by the addition of the HiBiT tag, these findings are consistent with streptococcin B being more efficiently secreted from the cell. These findings suggest that streptococcin B plays a significant and early role in competence-mediated fratricide.

### Streptococcin B inhibits *scbBC* expressing strains in plates and biofilms

**Fig 6:**
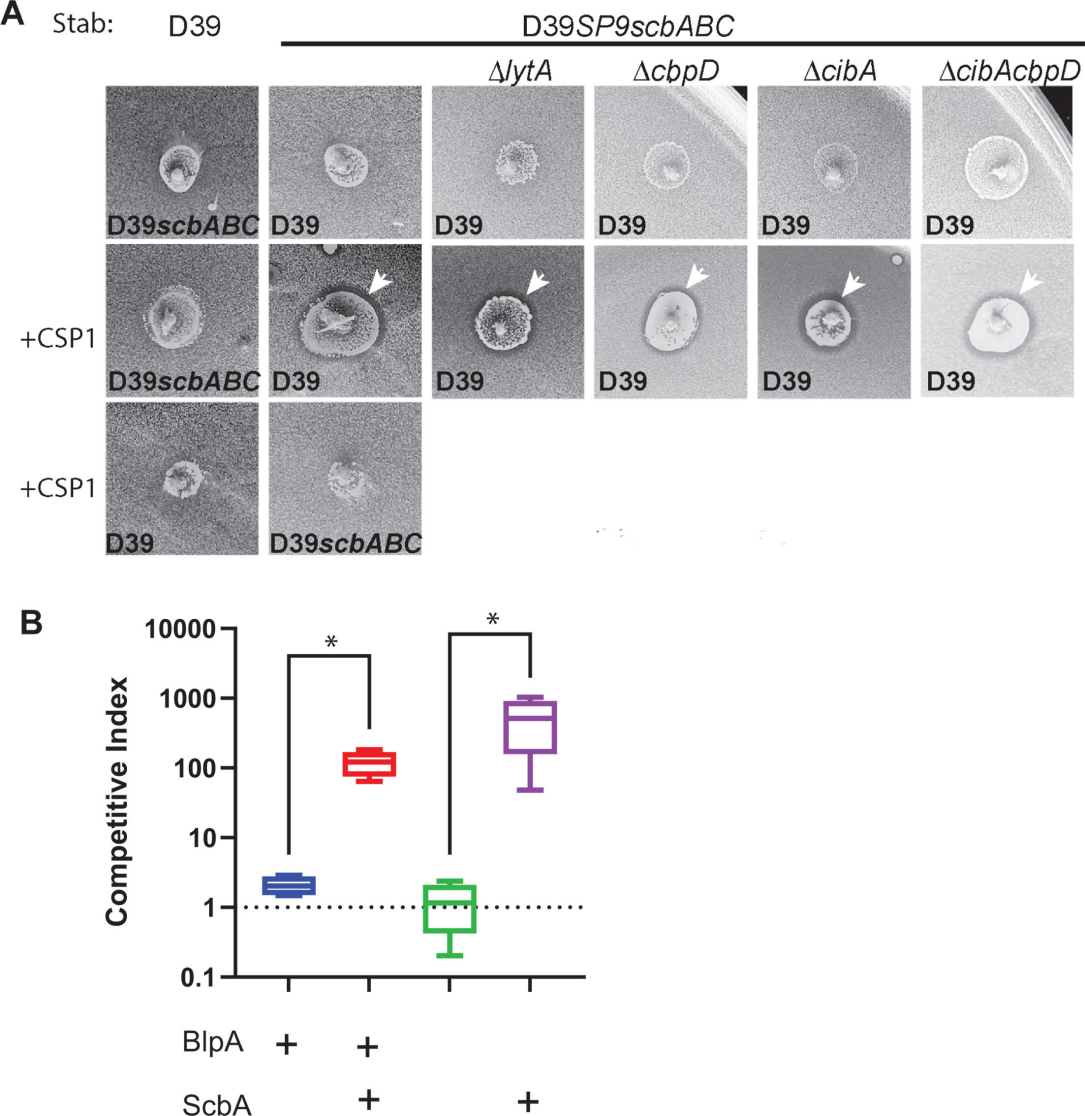
SP9-like *scbABC* expression results in phenotypic inhibition of D39 *scbBC* expressing strains in plate and biofilm assays. (**A**) Overlay assays demonstrating the ring of inhibition noted in *scbABC* containing strains following the addition of CSP. White arrows demonstrate the region of inhibition. The candidate producer strain is shown above the photos and the overlay lawn strain is marked on each individual photo. (**B**) Competitive index comparing the spectinomycin-resistant strain with either SP9 like *scbABC* or D39 like *scbBC* with a kanamycin/streptomycin otherwise matched competitor strain following overnight incubation in CDM at pH 7.4 over a fixed epithelial cell monolayer. *P < 0.05 by Mann-Whitney.

To determine if streptococcin B production produces detectible inhibition *in vitro*, we performed overlay assays using the SP9BS68 wild-type strain, D39 and D39 with the *sbcBC* locus replaced with the SP9 *scbABC* locus. No inhibition was noted in the absence of CSP addition; however, when CSP was added to the producer strain, SP9 and D39 expressing the SP9 version of *scbABC* both showed zones of inhibition when tested against D39 or D39Δ*scbABC* (Fig. 6A). Zones of inhibition could still be appreciated in Δ*lytA*, Δ*cibAB,* and Δ*cbpD* backgrounds, demonstrating that the inhibition noted was not the combined result of these fratricide effectors and rather related to expression of the *scbABC* locus alone.

To determine if inhibition of *scbBC*-expressing strains could be noted during growth in a biofilm, we created the *scb* variants in previously described D39 derivatives in which the native non-inhibitory *blp* locus found in D39 was replaced with a *blp* locus encoding the inhibitory pneumocins BlpIJK. To determine the role of enhanced competence activation in biofilm-mediated competition, isogenic versions of this strain were tested either with or without a frameshift mutation in *blpA*. We have previously described the behavior of these paired strains in *blp*-mediated biofilm competition (12). The competitor strains carry a spectinomycin cassette directly upstream of the *blp* locus and the sensitive D39 strain carries streptomycin and kanamycin resistance markers. Because all backgrounds are matched at the *blp* locus, no *blp* mediated competition is appreciated in these pairings. Equal inocula of the competitor and dual resistant strains were added to wells with a fixed epithelial substratum and allowed to establish overnight before plating. In static biofilm conditions, the strains carrying the SP9-like *sbcABC* had a competitive advantage compared with the D39-like *scbBC* in both the *blpA* sufficient and deficient backgrounds (Fig. 6B). This finding suggests that more permissive competence activation seen in the *blpA* sufficient backgrounds does not increase *scbABC*-mediated competition in this model.

## DISCUSSION

The pneumococcal population is characterized by significant diversity in genome content. There are at least 23 distinct bacteriocin or bacteriocin-like loci that are encoded in the pneumococcal chromosome, but only a handful of these have been characterized at the phenotypic level (9). Streptococcin B is in the family of lactococcin 972-like bacteriocins. Unlike traditional alpha-helical bacteriocins, this family forms a beta-sandwich that is not predicted to participate in pore formation (20). These bacteriocins have been shown to function by interacting with lipid II and inhibiting septum formation (21–23). The lipid II binding site appears to be separate from that of nisin. These bacteriocins are classically encoded in a three-gene operon that consists of a gene encoding the structural bacteriocin followed by two genes encoding presumed immunity and transport proteins, although experimental data for this are lacking.

Bacteriocins are often controlled by a quorum sensing system that restricts production to situations where the bacteriocin is likely to accumulate. Many classes of peptide-based quorum sensing systems have been described in *S. pneumoniae*, including the Tpr-Phr system and multiple Rgg-Shp class systems (9, 24). Here, we report for the first time the presence of a ComRS-type system in *S. pneumoniae*. ComRS systems were first described in *S. mutans* and *Streptococcus thermophilus* but have since been shown to be widespread throughout the streptococci. These systems have been demonstrated to regulate various aspects of bacterial physiology including the development of genetic competence and the production of bacteriocins (25–28). The ComRS system identified here in SP9 and D39-type strains of *S. pneumoniae* does not appear to be required for competence but does have the capability to regulate expression of the *scb* locus. Although the pneumococcal *scb* loci do not encode the *comS* gene, we show that the downstream ComR-XIP binding sites are intact and that ComR-dependent activation of the loci does occur upon addition of exogenous *S. pseudopneumoniae* SIP. Although this could be a vestigial phenotype, the widespread conservation of the *scb*-associated *scbR* regulator gene, its downstream ComR-XIP box containing the promoter, and the *scbBC* genes could indicate the system has retained evolutionary utility independent of its link to competence. *S. pseudopneumoniae* are inhabitants of the upper respiratory tract of humans and could be found in proximity to *S. pneumoniae in vivo* (29–31). The true prevalence of co-colonization has not been described. It is possible that the ability to express the *scbBC* genes thought to be involved in immunity in response to environmental SIP signaling from co-colonizing *S. pseudopneumoniae* strains provides both SP9 and D39-type *S. pneumoniae* strains with protection from resulting streptococcin B production. Should this be the case, it would represent an interesting interspecies interaction between *S. pneumoniae* and its poorly understood ecological competitor, *S. pseudopneumoniae*. Currently, experimental evidence confirming streptococcin B production by *S. pseudopneumoniae* is lacking, but the locus appears to encode all the elements needed for activation and secretion.

However, in most pneumococcal strains, the *scb* promoter region has gained a ComE biding site, allowing for coregulation and linking streptococcin B production to competence. In strains without the ComE binding site, endogenous activation of the locus presumably does not occur. Despite encoding intact immunity proteins, the lack of activation results in these strains being killed by streptococcin B encoding strains under conditions of competence induction. Given the prevalence of the *scbABC* locus in the pneumococcal population in over three-fourths of the genomes surveyed, this represents a previously unappreciated fratricide effector. The role of the *scbABC* locus as an early competence gene was not appreciated in early studies because the laboratory strains D39 and TIGR4, in which studies were performed, are both missing the ComE binding site. *scbABC* is produced at high amounts immediately following competence induction when compared with the kinetics of the *cibABC* operon. Additionally, because it is induced early and does not require a dedicated transporter, a greater proportion of peptide is secreted early during competence. Based on these observations, streptococcin B production is likely to play an equivalent or greater role in fratricide compared with known fratricide effectors, in both members of the population that have not induced competence or neighboring strains that lack the ComE binding site.

The pneumococcal population is divided into strains that either have or lack a functional BlpA transporter. BlpA intact strains not only activate the *blp* locus independently of competence, but the additional transporter allows for more permissive activation of competence (8, 14). Strains that have the ComE binding site in the *scbABC* promoter and an intact *blpA* were shown to activate the locus under a wider array of conditions than those that lacked BlpA expression. Despite this, the combination of an SP9-like *scbABC* and an intact *blpAB* is not greater than expected in the population based on the Boston genome collection. This suggests that selective pressure is not promoting this combination despite the competitive advantage noted *in vitro* possibly due to offsetting energetic cost. Recent publications have tied *blp* locus activation to a founder effect during colonization; however, this phenomenon was only described in a BlpAB deficient background (6). Because BlpAB-deficient strains can only activate the *blp* locus via competence activation, these findings suggest that other competence-regulated factors are also likely to play a role in early colonization fitness. Similarly, CibAB was shown to be required for blocking the new acquisition of pneumococcal isolates during early colonization using the infant mouse model (11). This work did not address the contribution of the streptococcin B locus to blocking new acquisition, but based on *in vitro* activity, it is likely that an advantage would be noted when competing a strain carrying a D39-like with a strain carrying a SP9-like locus.

As noted, the pneumococcal genome can encode up to five distinct lactococcin 972-like loci, encoding the bacteriocins streptococcins A–E. The regulatory controls of A, C, and D are unclear, but the promoters do not contain ComE binding sites. Streptococcin E, when found in a genome, replaces a different bacteriocin locus called Pneumolancidin G and based on its location would be predicted to be controlled by the TprA regulator and PhrA peptide pheromone. No function has been attributed to any of the non-B streptococcins. Based on the studies with streptococcin B, we expect that the other four loci provide a competitive advantage to expressing strains but potentially under different regulatory conditions. Some lactococcin 972-like homologs have been implicated in altering the immune response to infection. The *Streptococcus iniae* streptococcin B homolog has been shown to interfere with the respiratory burst in monocytes and preadministration of recombinant bacteriocin resulted in disseminated infection (32). The role of streptococcin B on the host immune response to infection has not been investigated.

## MATERIALS AND METHODS

### Growth conditions and strains used in this study

All strains were derived from either D39 or SP9BS68 as described. Strains are listed in Table S1, primers are listed in Table S2, and peptides are listed in Table S3. *S. pneumoniae* was grown in THY (Todd Hewitt medium supplemented with 5% yeast extract) at 37°C at pH 6.8 unless otherwise stated. For transformation, cells were grown in C + Y (pH 6.8) and stimulated for transformation in C + Y (pH 8.0) using 500 ng CSP1/mL. Following transformation, the cells were selected on tryptic soy agar supplemented with 5 μg/mL catalase and antibiotics as follows: streptomycin 100 μg/mL, kanamycin 500 μg/mL, spectinomycin 200 μg/mL, or medium containing 10% sucrose for counter selection of the Janus2 cassette. For natural induction assays, the cells were grown in CDM+ at the indicated pH and 330 μM firefly luciferin (88294; Thermo Fisher Scientific). The medium composition for CDM+ is described in reference 14. All synthetic peptides were ordered from Genescript at >95% purity. All assays were performed at least in triplicate on three separate occasions, and representative assays are shown.

### Strain construction

All reporter strains were constructed by PCR using a Pfusion (Termo), a high-fidelity polymerase, and HiFi Cloning (NEB) and confirmed by PCR and/or sequencing. Full details of strain construction can be found in the supplemental material.

### Peptide stimulation studies

Reporter strains were grown in CDM (pH 6.8) to OD_620_ = 0.2 in the presence of 330 μM luciferin and catalase. Additionally, 250 ng/mL of each peptide was added to the reporter strains at time 0. The plate was incubated in a Synergy HTX plate reader set to read absorbance at 620  nm and luminescence every 5  min at 37°C. Luminescence at the indicated times post peptide addition was compared with the same strain without peptide stimulation.

For kinetic assays, strains were inoculated in CDM at the indicated pH supplemented with catalase and luciferin using thawed starter cultures grown in THY at pH 6.8 to inhibit competence development. For these assays, 100 μL of starter culture was used per 5 mL of CDM. Both OD_620_ and luminescence were measured every 5 min during growth at 37°C and compared with blank lanes. To compare *scbABC* stimulation profiles at different pH levels in the *blpA* intact and non-intact backgrounds, the point at which the luminescence/OD value exceeded 250 was identified, and the OD_620_ at this point was recorded as an event as described by Wang et al. (14). Sixteen wells per strain were assayed with each experiment. Survival curves were created using these values to mark instances of locus stimulation. Wells that did not reach this threshold were right censored.

### Peptide secretion assays

Samples for extracellular peptide quantification were read with a Synergy HTX plate reader (Biotek) in a white 96-well plate (Costar, 3917) following the addition of HiBiT Extracellular Detection Reagent (Promega, N2421) at a 1:1 ratio. For endpoint assays, each sample was quantified with three technical replicates and read 5 min following reagent addition. For time-course assays, each sample was quantified with three technical replicates and read 1 min following reagent addition. Samples for intracellular peptide quantification were pelleted at 6,000 × *g*, 5 min, 4°C and resuspended in proteinase K buffer (20 mM MES [pH 6.5], 20 mM MgCl_2_, 0.5 M sucrose, 100 µg/mL proteinase K [Fisher Scientific, BP1700]). A 15 min incubation at 37°C removed residual extracellular peptide by proteinase K digestion. Afterward, proteinase K was inactivated by the addition of 1 mM phenylmethanesulfonyl fluoride (Calbiochem, 7110). The cells were lysed by the addition of 1% Triton X-100 followed by incubation at room temperature for 15 min. The lysed samples were then mixed with HiBiT Extracellular Detection Reagent at a 1:1 ratio in a white 96-well plate and read with a Synergy HTX plate reader. Each sample was quantified with three technical replicates using dilutions of the HiBiT tag peptide alone to create a standard curve.

### Plate overlay assays

TSA plates supplemented with catalase were stabbed with a pipet tip dipped in a starter culture of the indicated strain grown in THY to OD_620_ = 0.4 and frozen in 15% glycerol. The stabs were allowed to grow for 6 h, and 500 ng of CSP1 was added to each stab in 5 μL. This was allowed to dry, and the plates were incubated for an additional hour at 37°C. A soft agar overlay of the overlay strain was applied by adding 100 μL of a thawed starter culture and catalase to 5 mL of TS plus 2 mL of 55°C TSA and carefully applying this over the top of the plate. The plates were read for inhibition the following day after incubation at 37°C.

### Biofilm assays

H292 cells were grown to confluence on four-chamber slides, fixed in formalin, and then rinsed with 4 changes of PBS. Fixed cells were inoculated with plate-grown strains resuspended to an OD_620_ of 0.050 in CDM at pH 7.4. Strains were inoculated with or without competition into individual wells and incubated at 34°C in 5% CO_2_. Half of the cell volume was removed after 6 h of incubation and replaced with a fresh medium. Supernatants were removed from biofilms after overnight incubation, and the biofilm bacteria were harvested in 200 μL PBS using a small cell scraper. Dilutions were plated, and the plates were incubated in 5% CO_2_ at 37°C overnight before counting. All assays were performed on three separate occasions in at least four independently inoculated wells.

### Genome content

Using the PubMLST database, the isolates in the Boston collection were searched via BLAST for full-length BlpA, ScbA, ScbB, and CSP type (33). Isolates with either missing BlpA sequence data or typical truncated sizes were marked as BlpA non-intact. Any isolate with unique patterns was investigated manually for genome content. The phylogenetic tree was created using the Newick file from Mitchel et al. and displayed using iTOL.

## SUPPLEMENTAL MATERIAL

Supplemental material mbio.02993-24-s0001.pdf Supplemental methods, Figures S1 and S2, and Tables S1 to S3.

ASM does not own the copyrights to Supplemental Material that may be linked to, or accessed through, an article. The authors have granted ASM a non-exclusive, world-wide license to publish the Supplemental Material files. Please contact the corresponding author directly for reuse.
