# Supplementary material for: The pneumococcal bacteriocin streptococcin B is produced as part of the early competence cascade and promotes intraspecies competition
Source: mBio. 2024 Dec 17;16(2):e02993-24. doi: 10.1128/mbio.02993-24 (PMC11796350; doi:10.1128/mbio.02993-24)
Supplement: Supplemental material — Supplemental methods, Figures S1 and S2, and Tables S1 to S3. [file mbio.02993-24-s0001.pdf]

S1. Supplemental methods: Strain construction details.

The first reporters made were constructed to have either the *blp* promoter driving a HiBiT tagged version of the bacteriocin BlpI followed by the luciferase gene or the *cibA* promoter region driving a C-terminal HiBiT tagged version of CibA followed by the luciferase gene. These strains were created using a synthetic fragment that created the promoter/ bacteriocin HiBiT fusion and was designed to replace the janus 2 cassette in the transcriptionally silent CEP locus derived from strain 2779 (D39-CEP-Janus2-*luc*). CibAHiBiT was created using a synthetic 992bp fragment created by gBlocks Gene Fragments (IDT) that contains the upstream CEP region, the *cibA* promoter, the CibA ORF with a 10AA linker followed by the HiBiT tag GGDGGGGSGGGGSVSGWRLFKKIS followed by a portion of the luciferase gene in an operon format (sequence listed as primer 5 and 6). The synthetic region was joined to an up and downstream fragment created using primers 1-4. All subsequent fusions were created by amplifying the HiBiT tag/luciferase region of this strain with primers 7 and 8 and fusing via Gibson assembly to a variety of promoters that preceded a peptide of interest. Each peptide carries the same C terminus of a 10AA linker followed by the HiBiT tag. For SPD_145, SPD_0939, SPD_1518, SPD_1745 and SP_1786 region fusions, peptide reporter strains, primers 9-18 were used to place the quorum sensing promoters in front of a luciferase gene in the transcriptionally silent CEP locus by exchanging the constructed fragment into strain 3406 or 3404 which are D39 derivatives with either a disrupted or intact version of the *blpA* gene, respectively and with a janus2 locus in the CEP region. The promoter/peptide regions were all amplified from D39 except *scbA* which was cloned from the SP9BS68 genome. Clones were confirmed by PCR and phenotypic responsiveness to respective peptides. Streptococcin B strains were additionally confirmed by sequencing. The P*scbA* reporters were similarly constructed without a HiBiT tagged version of Streptococcin B arranged in an operon format with the luciferase gene from D39 or SP9BS68. The upstream region of the CEP locus was amplified using primers 1 and 2 and joined to the product of 17 and 19 or 17 and 20 to amplify the D39 or SP9BS68 promoters respectively and the downstream product of primers 21 and 4. The HiBiT +/- reporter strains behaved similarly in stimulation assays so the HiBiT tagged version was used to create the ComE binding site deletion. This deletion was created via Gibson assembly using Primers 17 and 22 and 23 and 4 and confirmed by sequencing to carry a deletion of the ComE binding site. The *scbABC* region from SP9BS68 was moved into the D39 strain replacing the native SPD_1786-1784 region as follows: The Janus2 cassette was inserted into the native *scbBC* (SPD_1785-6) region by Gibson assembly using primers 24/26 and 25/27 to create the 5'and 3' fragment and fusing these to the Janus2 cassette followed by reamplification with primers 28 and 29 transformation into the D39 strains. The cassette was exchanged by transforming with the *scbABC* region of SP9BS68 created with primers 24 and 27. A deletion of the upstream ComR (SPD_1786) and the scbBC region was created by transforming the Janus2 containing strain with a deletion made with primers 30/31 fused to 25/27. A deletion of just the *scbBC* region was created by transforming the Janus2 containing strain with a deletion made with primers 24/30 joined to 33/25. These strains were confirmed by PCR. The SP18BS74 allelic variant of *comR* was moved into the Janus2 containing strain using genomic DNA from SP18BS74 and selected on sucrose. The construct was back transformed into the Janus2 strain to

remove unlinked DNA. The correct insertion was verified using PCR with primer 56 and 19, primer 56 will only anneal to a unique region of the *comR* gene. The *amiCD* deletion was created by moving the *amiCD::janus2* deletion created in Wang et al [8] into the *scbA* reporter strain using primers 34/35. An in-frame, unmarked deletion was created by exchanging this region with the deletion of the *amiCD* region created by Wang et al using the same primers. The *comA*::janus deletion was moved into the Streptococcin B HiBiT expressing stain by transformation with genomic DNA from strain 2898 and selected for on kanamycin and confirmed by PCR.

cibAB, *lytA* and *cbpD* deletion strains were created by replacing the entire *cibAB*, *lytA* or *cbpD* ORF with either the spectinomycin (*cibAB*, *lytA*) or kanamycin (*cbpD*) resistance cassette using Gibson assembly. The individual pieces were made with primers 38-55. Fragments were re-amplified and inserted into the indicated strains by transformation. Deletions were confirmed by PCR.

Exposure to A549 cells. A549 cells were grown to confluence in half of a 96 well black tissue culture treated plate to confluence in DMEM/F12 with GlutaMAX (Gibco™) plus 10%FCS in 5% CO_2. No antibiotics were used. The cells remained at confluence for 10 days with q4 day media change. Just before the assay, monolayers were washed twice with PBS. Strain 3735 was grown to an OD_{620} of 0.3, pelleted and the pellet resuspended in RPMI with 1% FCS and 330 μM luciferin. The culture was incubated to allow for uptake of luciferin for 20 minutes at 37°C. The bacterial cells were added to the plate and the plate was spun at 2000 x g for 5 minutes at 4°C. The plate was read and then incubated in 5% CO_2 at 37°C. The plate was read for luminescence and OD_{620} at 30 and 60 minutes.

SP9BS68 *scbA* promoter region

...TTAATGATTATCTATCGTGTTTACAAGAAATTATGGATAAAACTCAAGATTATCAAAAGAAAC
CTCTTGTATTTATGTTTTTGTGGAAGCAAGCATTAAGAGTAGAAAGAGATTTTAGTTTAGCTGA
ATCATTTTATCAGTCTTCTAAAACATTTGCGAAGCTAATTGGAGATGAATTTCTAGTAAAGAAA
TTGACAGAGGAATGGCAAGAGGATGTCAAAAAATATTTATAAACATAGTGAATCAGTGACAAAA
ATGTCCTTGTCCTCGTATCAAAACAGTTCTAAAGTTCGTCTTTAGAACTGTTTTTTTAGATATA
AGCTAAAAATAATATGAAATAGTTAGATTTTAAGGACATTGATGTCCTTGATAGT **[GTGATAAAG**
ATGGAGTATAATGACTATAAAAGTTCATTTCAGGGTGCTATTTTGACAATTTAGAGAAAAACAAATAATT
TTAGAATATCATTTGGTAATATAATAGTATCAACGTTGATAATGGAAAGAGGAGCAAGTAACTCAACAAT
CTTGTAATCAAAAATAGAGGAGTATCAATATGAAACAAACAGTTAAAAAGTTAGCTCTTGTAGCGAGCAT
TGCAGCAACATTAGGTGGAGGTGTATCAGTTGCCTCTGCAGCCGTTCAATACCCAGAAGGTGGTGTTTGG
ACTTATGGTTCAGGTAACGGTGGTGCTTACTCAAACTACTATCACCCTTCAAAATACCATAGTTCAACAG
TTGTTAGCAGAAAAACTGGTTCATCTGACAAGGGATATGCTGGTGCTGGAGGACTTCTCGTGCATGGAT
TCGTACTTCTTGGGGAGAGAAAGTTGCATTCTACTATAATGTTTAGAATGGTATAGCCCTATTGTTTAGC
TTTACT] TGGTCACCTTCTCACTAAGTGACCAAGTAACTTTTTGGAGGAAATGATGAAACGTTTA
TTTATTTTGATTTCAATGGTATTGGTATCGCTTTATATGGTGATAACTTCCGTTGACCATCGAG
AAGAGATTTTATTTGGTAACTATCCTTCTGTTGATG...

Promoter region cloned

[Missing from D39]

scbR

comS?

ComR$_{BS}$

ComE$_{BS}$

scbA

scbB

D39 *scbB* promoter region

...TTAATGATTATCTATCGTGTTTACAAGAAATTATGGATAAAACTCAAGATTATCAAAAGAAAC
CTCTTGTATTTATGTTTTTGTGGAAGCAAGCATTAAGAGTAGAAAGAGATTTTAGTTTAGCTGA
ATCATTTTATCAGTCTTCTAAAACATTTGCGAAGCTAATTGGAGATGGATTTCTAGTAAAGGAA
TTGACAGAGGAATGGCAAGAGGATGTCAAAAAATATTTATAAACATAGTGAATCAGTGACAAAA
ATGTCCTTGTCCTCGTATCAAAACAGTTCTAAAGTTCGTCTTTAGAACTGTTTTTTTAGATATA
AGCTAAAAATAATATGAAATAGTTAGATTTTAAGGACATTGATGTCCTTGATAGTT **[CTTGAAA**
AAACGGATATAATGTATTTGAAGATGTTGCTAGCAGAAAGAACTTAATAAGCTTCTTAACATCG
CAGTGACTG] TGGTCACCTTCTCACTAAGTGACCAAGTAACTTTTTGGAGGAAATGATGAAACG
TTTATTTATTTTGATTTCAATGGTATTGGTATCGCTTTATATGGTGATAACTTCCGTTGACCAT
CGAGAAGAGATTTTATTTGGTAACTATCCTTCTGTTGATGTGACAGGAATG...

Promoter region cloned

[Missing from SP9]

comS?

ComR$_{BS}$

scbB

S. pseudopneumoniae scb promoter region

TTAATGATTATCTATCGTGTTTACAAGAAATTATGGATAAAACTCAAGATTATCAAAAGAAACC
TCTTATATTTATGTTTTTGTGGAAGCAAGCATTAAGAGAAGAAAGAGATTTTAGTTTAGCTGAA
TCATTTTATCAGTCTTCTAAAACATTTGCGCAGCTAATTGGAGATGAATTTCTAGTAAAGAAAT
TGACAGAGGAATGGCAAGAGGATGTCAAAAAATATTTATAAACATAGTGAATCAGTGACAAAAA
GGTCCCTGTTCTCGCATCAAAACAGTTCTAAAGTTCGTCTTTAGAACTGTTTTTTTAGATATAA
GCTAAAAATAATATGAAATAGTTAGATTTTAAGGACATTGGTGTCCTTG **[GAGTTTATTGATAT**
AAGTTTTATAATAGATGACATAGAAATAAGGAGGTGTGATATGTTTGGTTTTATCATGTTTTTA
ACATATTTTAGTTTTGGAGATTGGTGGCACGGATAGAAAGGAGAATGTAATGAGGTGTTGCTTT
GAAAAGGTCTATAATCCATGACTTAGTTTTACTTGAATAGAAAATAGACAATATATAATAAATA
TTTATGA] TATAATAAAAAATAGAGGAGTATCAATATGAAACAAACAGTTAAAAAATTAGCCCT
TGTAGCGAGTCTTGTAGCAACATTAGGTGGAGGAGTAGCAGTAGTATCGGCCGCGGTTAAGTAT
CCAGGTGGTGGTGTCTGGACGTATGGTTCGGCTAACGGAGGTGCTTACTCAAACTACTATCACC
CCTCAAAATACCATAGTTCAACAGTTTCTAGCAGATGGAATAGTTCATCTGATAAGGGATATGC
TGATGCAGGAGGAACATCTCGAGCTTGGATTCGTACTGCGTGGGGCGAGCAAGTAAGCTTTTAT
TATGATTATTAAAAATTATACACGAGAAAGTAAAAACATTTCTGGGTGAGTTGATAACAATGCT
TTACTTGGTCATCTCTCTAGCAGGTGACCAAGTAACTTTTTGGAGGAAATGATGAAACGTTTAT
TTATTTTGATTTCAATGGTATTGGTATCGCTTTATATGGTGATAAATTCCGTTGATCATCGAGA
AGAGATTTTATTTGGTAACTATCCCTCTGTTGATGTGATGGGTACT...

[Missing from SP9]

scbR

ComR$_{BS}$

comS

scbA

scbB

Supplemental Figure 1. **Annotated sequence of the SP9, D39 and *S. pseudopneumoniae scb* promoter regions between the *scbR* and *scbB* genes.** The end and beginning of the *scbR* and *scbB* genes are highlighted in brown or red, respectively. *scbA*, which is only present in SP9 and *S. pseudopneumoniae* is highlighted in blue. The ComR binding site and the ComE binding site are highlighted in yellow or pink, respectively. The promoter regions that were cloned 5’ to a luciferase gene are underlined. The hypothetical *comS* that was used to derive a non functional XIP peptide is shown in light blue. The *comS (scbS)* that was used to derive the functional SIP sequence from the *S pseudopneumoniae* locus is highlighted in green. The bracketed regions in bold in each sequence are unique to that promoter region.

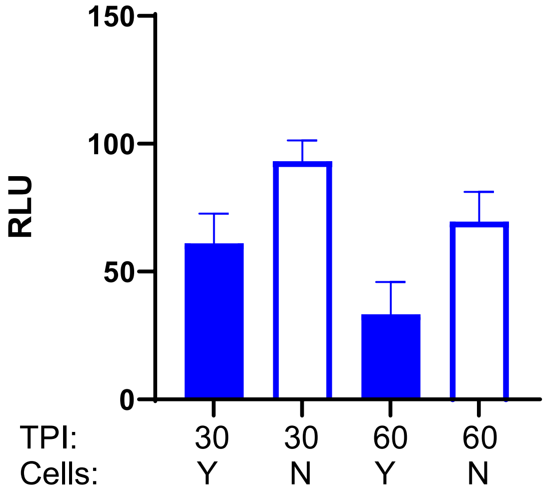

Supplemental Figure 2. Exposure to live A549 cells does not induce the D39 *scbBC* promoter. Reporter strain 3735 with the D39 *scbBC* promoter driving luciferase production was incubated with live A549 cells at 5% CO_2 in RPMI + 1%FCS for the indicated times in the presence of luciferin. RLU was determined in wells with and without cells. TPI= Time post infection. Assay was done with 6 replicates for each condition and was repeated once for verification. A representative assay is shown.

Table S1 Strains used in this study

Strain number	Background	Mutation	Properties	Reference
641	D39	none		1
299	D39	D39, *aad9* upstream of *blpT*, *SpeR*	D39 with native disrupted *blpA*	2
300	D39	blpP164, *aad9* upstream of *blpT, SpeR*	D39 with active *blp* locus from strain P164	2
309	D39	PSD300 with *blpAfs* mutation	D39 with active *blp* locus from strain P164 with *blpA* frameshift mutation	2
799	SP9BS68	none		3
4016	D39	300 but *CEP::pscbABCluc*	D39 with active *blp* locus from strain P164, $PsbcA_{SP9}$ reporter in CEP locus	this study
3493	D39	300 but $CEP::pscbA_{HibiT}luc$	D39 with active *blp* locus from strain P164, *sbcA* reporter with *scbAHiBiT* tag in CEP locus	this study
3735	D39	300 but $CEP::pscbBC_{D39}luc$	D39 with active *blp* locus from strain P164, $PsbcB_{D39}$ reporter in CEP locus	this study
4015	D39	299 but *CEP::pscbABCluc*	D39 , $PsbcA_{SP9}$ reporter in CEP locus	this study
3489	D39	299 but $CEP::pscbA_{HibiT}luc$	D39, *sbcA* reporter with *scbAHiBiT* tag in CEP locus	this study
3613	SP9BS68	799 but $CEP::pscbA_{HibiT}luc$	SP9, *sbcA* reporter with *scbAHiBiT* tag in CEP locus	this study
3418	D39	299 but $CEP::pblpI_{HiBiT}luc$	D39 , *PblpI* reporter with *BlpIHiBiT* tag in CEP locus	this study
3420	D39	299 but $CEP::pcibA_{HiBiT}luc$	D39 , *PcibA* reporter with *CibAHiBiT* tag in CEP locus	this study

3453	D39	299 but *CEP::p144*$_{HiBiT}$*luc*	D39 , *PSD_0145* reporter with *SHP144HiBiT* tag in CEP locus	this study
3455	D39	299 but *CEP::p939*$_{HiBiT}$*luc*	D39 , *PSD_0939* reporter with *SHP939HiBiT* tag in CEP locus	this study
3459	D39	299 but *CEP::p1518*$_{HiBiT}$*luc*	D39 , *PSD_1518* reporter with *SHP1518HiBiT* tag in CEP locus	this study
3464	D39	299 but *CEP::p1745*$_{HiBiT}$*luc*	D39 , *PSD_1745* reporter with *SPD_1745HiBiT* tag in CEP locus	this study
3725	D39	300 but *CEP::prtgS-luc*	D39 , *PrtgR* reporter with *RtgR HiBiT* tag in CEP locus	this study
3757	D39	3493 but *ΔcomR-scbC*	D39 with active *blp* locus from strain P164, *sbcA* reporter with *scbAHiBiT* tag in CEP locus, deletion in upstream *comR* and *scbABC* locus.	this study
3759	D39	3493 but *ΔscbABC*	D39 with active *blp* locus from strain P164, *sbcA* reporter with *scbAHiBiT* tag in CEP locus, deletion *scbABC* locus.	this study
3878	D39	3493 but *CEP::pscbAΔcomE*$_{BSHiBiT}$*luc*	D39 with active *blp* locus from strain P164, *sbcA* reporter with *scbAHiBiT* tag in CEP locus with a deletion in the ComE binding site in the *sbcA* promoter.	this study
3898	D39	3493 but *ΔamiCD*	D39 with active *blp* locus from strain P164, *sbcA* reporter with *scbAHiBiT* tag in CEP locus but	this study

			with a deletion in the *amiCD* genes.	
3802	D39	300 but *scbABC*$_{SP9}$	D39 with active *blp* locus from strain P164 but with *scbABC* locus from SP9	this study
3832	D39	309 but *scbABC*$_{SP9}$	D39 with active *blp* locus from strain P164 with *blpA* frameshift mutation but with *scbABC* locus from SP9	this study
3771	D39	300 but *CEP::KnR, StR*	D39 with active *blp* locus from strain P164 but *rpsL*, *CEP::KnR*	this study
3900	D39	3489 but *comA::sJanus+*	D39, *sbcA* reporter with *scbAHiBiT* tag in CEP locus and *ΔcomA::sjanus+*	this study
3676	D39	641 but *scbABCSP9*	D39 with *scbABC* locus from SP9	this study
3870	D39	3676 but *ΔcibAB::spectR*	D39 with *scbABC* locus from SP9, deletion in *cibAB*	this study
3864	D39	3676 but *ΔlytA::spectR*	D39 with *scbABC* locus from SP9, deletion in *lytA*	this study
3868	D39	3676 but *ΔcbpD::knR*	D39 with *scbABC* locus from SP9, deletion in *cbpD*	this study
3872	D39	3676 but *ΔcibAB::spectRcbpD::kanR*	D39 with *scbABC* locus from SP9, deletion in *cibAB* and *cbpD*	this study
4081	D39	3493 but *comR-scbC* from SP18BS74	D39 with active *blp* locus from strain P164, *sbcA* reporter with *scbAHiBiT* tag in CEP locus and *comR-scbC* region from SP18BS74	this study

1 Avery, Macleod, and McCarty 1944 J.Exp Med 79:137-58. 2. Wholey et al Plos Pathogens 2016 12(2): e1005413, 3. Hiller et al (2007)Jbac 189: 8186-8195

Table S2
Primers/ synthetic fragments used in this study.

Primer	Sequence (5'-3')	Brief Description
1	TGGCTGACTAGGAGGAAGGA	Cep up F
2	ATTCCCATAAAAATTGACATGG	Cep up R
3	CCCGGTATCCAGATCCACAA	luc down F
4	AATCGTCGTATGCAGTGAAA	luc down R
5	GGTAGAAATCCGTCCAGGTCACTATGTTTGGGCGAACCAA<br>GCCGAATTAGCACGTTATCAAAAAGGACTAAACTAATAATG<br>GTTTTATAATTTCCATGTCAATTTTTATGGGAATTAGGCTCT<br>TTGTCAACTGTAGTGGGTTGAAGTCAGCTAAGCTCGAGAAA<br>GGACAAATTTCGTCCTTTCTTTTTTGATGTTCAAAGCGATAA<br>AAATCCGTTTTTTGAAGTTTTCAAAGTTCCGAAAACCAAAGG<br>CATTGCGCTTGATAAGTTTGATGAGATTATTGGTCGCTTCC<br>GTGATTAGAAATTATTTTTTTACTTTTCCTTTTCTTTTCCGAA<br>TATAAAAGTGAACAAGAAAAAAGGAGGAAAGTTCAATGACA<br>AATTTTGACATTCTTGACAATCAATTTTTATCCTTATCTGAAA<br>ATGAATTGTCAGATATTGATGGCGGTCTCGCTCCCTTGGTT<br>ATCTTTGGAGTAGCAGTATCTTGGAAGGCTATTGCAGGTGG<br>AACAGCACTTATAGGTTCTGGTTTGGCAGCTGGTTATTTTTT<br>AGGAGGAGATGGTGGTGGAGGTTCAGGAGGTGGAGGTTC<br>TGTTTCTGGTTGGCGTCTTTTTAAAAAAATTTCATAAAGGAG<br>GAAAATTAATGGAAGACGCCAAAAACATAAAGAAAGGCCC<br>GGCGCCATTCTATCCTCTAGAGGATGGAACCGCTGGAGAG<br>CAACTGCATAAGGCTATGAAGAGATACGCCCTGGTTCCTG<br>GAACAATTGCTTTTACAGATGCACATATCGAGGTGAACATC<br>ACGTACGCGGAATACTTCGAAATGTCCGTTCGGTTGGCAG<br>AAGCTATGAAACGATATGGGCTGAATACAAATCACAGAATC<br>GTCGTATGCAGTGAAAACTCTCTTCAATTCTTTATGCCGGT<br>GTTGGGCGCGTTATTTATCGGAGTTGCAGTTGCGCCCGCG<br>AACGACATTTATAATGAACGTGAATTGCTCAACAGTATGAA<br>CATTTCG	cibA Hibit insertion

6	ACCAAGCCGAATTAGCACGTTATCAAAAAGGACTAAACTAA TAATGGTTTTATAATTTCCATGTCAATTTTTATGGGAATTAG GCTCTTTGTCAACTGTAGTGGGTTGAAGTCAGCTAAGCTCG AGAAAGGACAAATTTCGTCCTTTCTTTTTTGATGTTCAAAGC GATAAAAATCCGTTTTTTGAAGTTTTCAAAGTTCCGAAAACC AAAGGCATTGCGCTTGATAAGTTTGATGAGATTATTGGTCG CTTCCGTGATTAGAAATTATTTTTTTTAAACATTTAGAGGTG GCTTGAAATAAAAAAGCTAATTCAAGACGTTTCGATGCCAA TTCAAGATTTGGATGAAAAAAATTAAAAAATAATGATATACT AAACTTGTCAAAGTTGCAACAAGACAAAAATTAAAAATAAAA AAGGAGTATTTGTCATGAATACAAAAATGATGTCACAATTTT CTGTTATGGATAATGAAGAACTCGAAATAGTTAGTGGAGGA AGAGGCAATTTAGGATCTGCAATCGGTGGTTGTATTGGAG CAGTACTATTAGCTGCTGCGACTGGTCCGATAACTGGGGG AGCAGCAACACTTATTTGTGTAGGTTCAGGAATTATGTCCT CTTTGGGTGGTGGAGGTTCAGGAGGTGGAGGTTCTGTTTC TGGTTGGCGTCTTTTTAAAAAAATTTCATAAGGAGGAAAATT AATGGAAGACGCCAAAAACATAAAGAAAGGCCCGGCGCCA TTCTATCCTCTAGAGGATGGAACCGCTGGAGAGCAACTGC ATAAGGCTATGAAGAGATACGCCCTGGTTCCTGGAACAATT GCTTTTACAGATGCACATATCGAGGTGAACATCACGTACGC GGAATACTTCGAAATGTCCGTTCGGTTGGCAGAAGCTATGA AACGATATGGGCTGAATACAAATCACAGAATCGTCGTATGC AGTGAAAACTCTCTTCAATTCTTTAT	blpI Hibit insertion
7	GGTGGTGGAGGTTCAGGAGG	peptide link Hibit F
8	GGAAGCGACCAATAATCTCATC	CEP up R
9	TGAGATTATTGGTCGCTTCCTTAAAAATGGAATGACAATAA CCCACTC	SPD_145 with promoter_fwd
10	CCTCCTGAACCTCCACCACCTCTTCTACGGCGGCCAAAC	SPD_145 with promoter_rev
11	TGAGATTATTGGTCGCTTCCAGTTTTCCCATTTTCCCAAC	SPD_939 shphibit_fwd
12	CCTCCTGAACCTCCACCACCTCCACCAACAATAATGATAAT ATC	SPD_939 shphibit_rev
13	TGAGATTATTGGTCGCTTCCATTGTTCCTCCTAGAAAAATG	SPD_1518 with SHP_fwd
14	CCTCCTGAACCTCCACCACCTCCCCAAAACCATGTTTC	SPD_1518 with SHP_rev
15	TGAGATTATTGGTCGCTTCCTAAAGATAATAAACCTTCCTAT TTGCTTAATTTC	SPD_1745_fwd
16	CCTCCTGAACCTCCACCACCATCCGCCTTCCCAACATC	SPD_1745_rev
17	TGAGATTATTGGTCGCTTCCACATAGTGAATCAGTGACAAA AATG	SPD_1786_fwd
18	CCTCCTGAACCTCCACCACCAACATTATAGTAGAATGCAAC TTTC	SPD_1786_rev scbA hibit
19	ATGTTTTTGGCGTCTTCCATCATTTCCTCCAAAAAGTTAC	*scb* D39 promoter reverse

20	CGTCTTCCATATTGATACTCCTCTATTTTTGATTACAAG	*scb* prom R
21	ATGGAAGACGCCAAAAACATAAAG	promoter luc down_F
22	TTTGTTTTTCCTTTTATAGTCATTATACTCCATCTTTATC	scbAhibit ΔcomE up_rev
23	ACTATAAAAGGAAAAACAAATAATTTTAGAATATCATTTG	scbA ΔcomeE down_fwd
24	TTAATTGGTCAAAAAATTAAAGAGATTC	1786 up_fwd
25	GGAATGCCACTCTCAATC	1786 down_rev
26	TCCATTAAAAATCAAACGGAGGACATCAATGTCCTTAAAAT C	1786 up_rev janus
27	AGGATCCCTCCAGTAACTCGAGGAAACTTGCTCATGTG	1786 down_fwd janus
28	GAGATGAGCAAGAACTGACAG	1786 up_fwd nested
29	GTAGTAAGGCAAATTAGCCACT	1786 down_rev nested
30	CAAGTTTCCTGGACATCAATGTCCTTAAAATC	1786 up_rev IF
31	CTGGAAGATACCTATGAGATTATC	in frame comR ko up_fwd
32	CAAGTTTCCTTAACATCAGACGCCTCCTC	in frame comR ko up_rev
33	ATTGATGTCCAGGAAACTTGCTCATGTG	1786 down_fwd IF
34	GCCTTGAATTTTGCTCTTGATCG	CEP up F
35	CCTTCCTCCTAGTCAGCCAG	amiE R
36	AATCGTCGTATGCAGTGAAA	CEPluc R
37	ATTCCCATAAAAATTGACATGG	CEP up R primer
38	CGCTCAGAATATTGGGACTAAG	kan insertion in cbpD
39	TCAAACGGATCCTCTTGCTATAAACGGTAAAATTTTC	kan insertion in cbpD
40	GCAAGAGGATCCGTTTGATTTTTAATGGATAATG	kan insertion in cbpD
41	CTCCAATTTTGGAATTCTAGGTACTAAAACAATTCATC	kan insertion in cbpD
42	TAGAATTCCAAAATTGGAGTAGGAGAAATTTCCTGCTC	kan insertion in cbpD
43	TTGCCAAGGGTTTGCTCGCA	kan insertion in cbpD
44	GCCCCATCATAGGCCAGAGC	spectR insertion in cibAB
45	TTCTAGAGCTGAACTTTCCTCCTTTTTTCTTGTTC	spectR insertion in cibAB
46	GAAAGTTCAGCTCTAGAACTAGTGGATCCC	spectR insertion in cibAB
47	TGTTTCTTATCAATTTTTTTATAATTTTTTTAATCTG	spectR insertion in cibAB

48	TAAAAAAATTGATAAGAAACACATTTTTAGAAGGATAAATTT TATTG	spectR insertion in cibAB
49	GCAATTTGTCAGACAAGAGTTC	spectR insertion in cibAB
50	GGGCTTGATGAGTTCAATTG	spectR insertion in lytA
51	TTCTAGAGCATTCTACTCCTTATCAATTAAAACAAC	spectR insertion in lytA
52	GAGTAGAATGCTCTAGAACTAGTGGATCCC	spectR insertion in lytA
53	CATTCCATTACAATTTTTTTATAATTTTTTTAATCTG	spectR insertion in lytA
54	TAAAAAAATTGTAATGGAATGTCTTTCAAATC	spectR insertion in lytA
55	GGTATCCATCATTCCTCAATC	spectR insertion in lytA
56	ATGAGCAACTTCCTAATGAGG	unique comR verify

Table S3
Peptides used in this study.

**Peptides**	**Sequence**
CSP1	ENRKSJFFRDFUKQRKK
BlpC2	GWWEELLHETILSKFKITKALELPIQL
SHP144	EWVIVIPFLTNL
SHP939	DIIIIVGG
SHP1518	LIWFETWFWG
PhrA	SNGLDVGKAD
S.pseudopneumoniae SIP	GDWWHG
RtgS	AIIFPWGWP
S. pneumo hypo XIP	SYQNSSKVR
